# Titanium dioxide nanoparticles oral exposure to pregnant rats and its distribution

**DOI:** 10.1186/s12989-019-0313-5

**Published:** 2019-07-18

**Authors:** Jinsoo Lee, Ji-Seong Jeong, Sang Yun Kim, Min-Kyu Park, Sung-Deuk Choi, Un-Jung Kim, Kwangsik Park, Eun Ju Jeong, Sang-Yoon Nam, Wook-Joon Yu

**Affiliations:** 1 Developmental and Reproductive Toxicology Research Group, Korea Institute of Toxicology, Deajeon, 34114 Republic of Korea; 2 College of Veterinary Medicine, Chungbuk National University, Cheongju, Republic of Korea; 3 School of Urban and Environmental Engineering, Ulsan National Institute of Science and Technology, Ulsan, Republic of Korea; 4 UNIST Environmental Analysis Center (UEAC), Ulsan National Institute of Science and Technology, Ulsan, Republic of Korea; 5 College of Pharmacy, Dongduk Women’s University, Seoul, Republic of Korea

**Keywords:** Titanium dioxide nanoparticles, Developmental toxicity, Maternal and fetal distribution, Nanotoxicity

## Abstract

**Background:**

Titanium dioxide (TiO_2_) nanoparticles are among the most manufactured nanomaterials in the industry, and are used in food products, toothpastes, cosmetics and paints. Pregnant women as well as their conceptuses may be exposed to TiO_2_ nanoparticles; however, the potential effects of these nanoparticles during pregnancy are controversial, and their internal distribution has not been investigated. Therefore, in this study, we investigated the potential effects of oral exposure to TiO_2_ nanoparticles and their distribution during pregnancy. TiO_2_ nanoparticles were orally administered to pregnant Sprague-Dawley rats (12 females per group) from gestation days (GDs) 6 to 19 at dosage levels of 0, 100, 300 and 1000 mg/kg/day, and then cesarean sections were conducted on GD 20.

**Results:**

In the maternal and embryo-fetal examinations, there were no marked toxicities in terms of general clinical signs, body weight, food consumption, organ weights, macroscopic findings, cesarean section parameters and fetal morphological examinations. In the distribution analysis, titanium contents were increased in the maternal liver, maternal brain and placenta after exposure to high doses of TiO_2_ nanoparticles.

**Conclusion:**

Oral exposure to TiO_2_ during pregnancy increased the titanium concentrations in the maternal liver, maternal brain and placenta, but these levels did not induce marked toxicities in maternal animals or affect embryo-fetal development. These results could be used to evaluate the human risk assessment of TiO_2_ nanoparticle oral exposure during pregnancy, and additional comprehensive toxicity studies are deemed necessary considering the possibility of complex exposure scenarios and the various sizes of TiO_2_ nanoparticles.

## Introduction

Nanotechnology is a rapidly growing field in recent decades and is widely applied in various areas of industry [1]. The use of nanotechnology extends to cosmetics, fabrics and clothing, personal care items, cleaning solutions, sporting equipment and electronics as well as toys for children [2, 3]. Nanomaterials comprise natural, incidental or manufactured material-containing particles with one or more external dimensions in the size range of 1 nm – 100 nm [4]. The size-dependent properties of nanomaterials increase the surface-to-interaction, the possibility of improper interactions with intracellular components and unusual electronic properties, such as electron donation or acceptance [5]. These characteristic properties raise concerns regarding the potential health risk to humans and livestock, as well as the environment [6, 7].

Titanium dioxide (TiO_2_) nanoparticles are also widely used nanomaterials and are among the top five nanomaterials used in consumer products [8]. TiO_2_ nanoparticles are used in paints, coatings, plastics, papers, inks, medicines, pharmaceuticals, food products, cosmetics and toothpastes [9–11]. The constant use of TiO_2_ nanoparticle-containing products increases the possibility of chronic exposure and accumulation in the internal organs of humans. In particular, oral and respiratory exposures are considered the most prevalent exposure routes to humans [12]. Oral exposure is an important route for absorption because water, liquid beverages and drug carriers may contain TiO_2_ nanoparticles [13]. When TiO_2_ nanoparticles (25, 80, and 155 nm; 5 g/kg; single oral dose in mice) enter the circulatory system via oral exposure, they are retained in the internal organs [14].

Several toxicity studies with TiO_2_ nanoparticles have been recently conducted; however, there is little toxicological information on TiO_2_ nanoparticle exposure during pregnancy. Shimizu et al [15] reported that subcutaneous exposure to TiO_2_ nanoparticles (2570 nm; 100 μl suspended at 1 μg/μl) during gestation (gestation days [GDs] 6, 9, 12 and 15) in ICR mice induced changes in gene expression related to brain development, cell death, response to oxidative stress, and mitochondria in the brain during the prenatal period. Takeda et al. [16] reported that subcutaneous exposure of ICR mice to TiO_2_ nanoparticles (25 and 70 nm; 16 mg/kg) during gestation (GDs 3, 7, 10 and 14) induced postnatal reproductive toxicities in male offspring, including disrupted seminiferous tubules and tubule lumens with few mature sperm, decreased sperm production and epididymis sperm motility. In addition, TiO_2_ nanoparticles were detected in cells of the olfactory bulb and cerebral cortex in these postnatal animals. These previous studies indicated that TiO_2_ nanoparticle exposure during pregnancy is able to induce toxic effects. However, the opposite result was also reported: oral exposure of six types of TiO_2_ particles, including pigment grade and nanoscale (42, 43, 47, 153, 195 and 213 nm; 100, 300, and 1000 mg/kg; daily, beginning on GDs 6 through 20 in rats), did not induce maternal and embryo-fetal developmental toxicities [17].

The objective of this study was to confirm the maternal and embryo-fetal toxicities of orally exposed TiO_2_ nanoparticles during pregnancy. In addition, we also analyzed the internal concentration of titanium in maternal and fetal tissues. The results of this study will contribute to elucidating the potential effects of TiO_2_ nanoparticles on humans and support the accurate risk assessment of these nanoparticles at different sizes and under complex exposure scenarios.

## Materials and methods

### TiO_2_ nanoparticles and physicochemical characterization

TiO_2_ nanoparticles were obtained from Evonik Industries (Germany) as a fine white powder with a hydrophilic characteristic caused by hydroxyl groups on the surface. The nanoparticles consisted of aggregated primary particles; the mean diameter of the primary particle was approximately 21 nm, and the weight ratio of anatase/rutile was approximately 80/20 according to the manufacturer’s information.

Physicochemical characterization of TiO_2_ nanoparticles was confirmed with an additional analytical method. The primary particle size and morphology were analyzed by a transmission electron microscope (JEM-2100F, JEOL, Japan) operating at 200 kV. TiO_2_ NPs for transmission electron microscope (TEM) analysis were deposited on carbon-coated nickel mesh grids and were air-dried overnight before analysis. The purity was also analyzed with energy-dispersive X-ray (EDX) analysis on the same TEM images (JEM-2100F TEM equipped with an X-Max^N^ 150 mm^2^ silicon drift detector, Oxford Instruments, UK). The average primary particle size was calculated by measuring at least 100 particles using an image analyzer program (DigitalMicrograph, Gatan Inc., USA). The hydrodynamic diameter and zeta potential of TiO_2_ nanoparticles in deionized water (10 mg/ml concentration) were analyzed by the dynamic light scattering (DLS) method (ELS-8000, Otsuka Electronics, Japan).

### Animals and experimental design

Nine-week-old specific pathogen free (SPF) female Sprague-Dawley rats were obtained (Orient Bio Inc., Republic of Korea) and permitted a 5-days period of acclimation to the animal room environment. Females were selected for mating on the basis of adequate body weight and freedom from clinical signs of disease or injuries during the acclimation period. Females were mated by placement in the cage of a male that was maintained only for mating without any treatment. Sixty-four mating-proven female rats were selected for this study. The day of sperm and/or vaginal plugs detection was designated as day 0 of gestation. Pregnancy was determined by confirmation of implantation sites on the uterus at the time of final sacrifice.

The animal room environment was automatically controlled according to institutional criteria (target range: temperature of 23 ± 3 °C, relative humidity of 30–70%, approximately 12-h light cycle with 150–300 Lux, and ventilation at 10–20 times/hour). A standard rodent pellet diet irradiated by gamma-ray (PMI Nutrition International, USA) was provided to the animals ad libitum. Titanium was not detected in the rodent pellet diet according to the chemical composition results from the supplier. The animals had ad libitum access to filtered, ultraviolet light-irradiated municipal tap water at all times. Aspen animal bedding material (Bio Lab, Republic of Korea) was sterilized and then provided to the animals in each cage. There were no known contaminants in the food, water and bedding at levels that would be expected to interfere with the results of the study.

TiO_2_ nanoparticles were suspended in deionized water for administration via the gastrointestinal route. To obtain a homogenized suspension, the dosing formulation was continuously stirred with a magnetic stirrer during the dosing procedure. TiO_2_ nanoparticles were administered by oral gavage to mated females to evaluate the potential maternal and embryo-fetal development toxicity of TiO_2_ nanoparticles. This study design refers to the OECD Guideline 414 (Prenatal Developmental Toxicity Study) [18] and was carried out in a good laboratory practice (GLP) facility but was not conducted within the scope of GLP regulations. In addition, we also analyzed the internal distribution of titanium in maternal and fetal tissues after repeated oral exposure during pregnancy. Twelve females per group in the toxicology group (total 48 females) and 4 females per group in the tissue distribution group (total 16 females) were used in this study. TiO_2_ nanoparticles were administered daily by oral gavage from GDs 6 to 19 at dose levels of 0, 100, 300 and 1000 mg/kg with a dose volume of 10 mL/kg.

All procedures with animals were in compliance with the Animal Protection Act of Korea and the Guide for the Care and Use of Laboratory Animals published by the Institute for Laboratory Animal Research (ILAR). The Korea Institute of Toxicology (KIT) received full accreditation from the Association for Assessment and Accreditation of Laboratory Animal Care International (AAALAC International) in 1998, which has been renewed regularly. This study was reviewed and assessed by the Institutional Animal Care and Use Committee (IACUC) of KIT.

### In-life maternal examinations

A mortality observation was conducted twice daily (once at the start of the animal room procedure and once at the end of the animal room procedure). Observation of general clinical signs, including general appearance and behavioral changes, were conducted twice a day during the treatment period (before and after dosing) and once a day during the nontreatment period. During pregnancy, maternal animals were especially monitored for signs of abortion or premature delivery. Body weights and food consumption were measured individually on GDs 0, 6, 9, 12, 15, 17 and 20.

### Cesarean section and fetal morphological examinations

On GD 20, all toxicology group females were euthanized using CO_2_ gas for macroscopic observation and cesarean section. All females were examined carefully for external, abdominal, thoracic and cranial cavity abnormalities. Special attention was paid to the organs of the reproductive system. Gravid uteri were retrieved and then weighed to calculate the corrected terminal weight (body weight on GD 20 minus gravid uterine weight) and the net body weight change (corrected terminal weight minus body weight on GD 6). The corpora lutea, implantation sites, live/dead fetuses and resorptions (early or late) were counted or measured, and then calculated pre-implantation loss, post-implantation loss and fetal death. Each live fetus was weighed and sexed. In addition, each placenta was weighed and examined macroscopically.

Fetal morphological examinations, including external, visceral and skeletal examinations, were conducted. Fetuses were numbered from the left uterine horn to the right uterine horn. Alternate fetuses were selected for either skeletal or visceral examination (odd numbers: skeletal examination, even numbers: visceral examination). Live fetuses retrieved from gravid uteri were examined immediately to evaluate external abnormalities. For fetal visceral examinations, fetuses were fixed with Bouin’s solution, and then modified Wilson’s method [19] for the head, Nishimura’s method [20] for the thorax and Staples’s method [21] for the abdomen were used. For skeletal examinations, fetuses were fixed with 70% ethanol, and then Dawson’s method [22] was used after staining with alizarin red. Fetal morphological abnormalities were classified as malformation or variations according to the severity of findings. In addition, we used the terminology suggested in an internationally developed glossary of terms for structural developmental abnormalities in common laboratory mammals [23].

### Tissue collection and preprocessing

On GD 20, all tissue-distribution group females were euthanized using CO_2_ gas to conduct the tissue collection. Maternal tissue collection (approximately 200 mg each), including liver (middle lobe), brain, and blood, was conducted. Fetal tissue collection (approximately 200 mg each), including liver, brain, blood and placenta, was conducted. At least 3 fetuses from a litter were used for fetal tissue collection, and collected samples were pooled by a litter. All collected samples were weighed to quantitatively calculate the tissue distribution and then maintained in frozen condition (approximately − 80 °C) until titanium content analysis.

For the evaluation of tissue levels of titanium, the samples were digested with a tri-acid mixture. The tri-acid mixture was prepared with concentrated hydrofluoric acid (HF, 49%, J.T. Baker, USA), nitric acid (HNO_3_, 60%, Matsunoen Chemical LTD, Japan), and hydrogen peroxide (H_2_O_2_, 30%, J.T. Baker, USA) mixed in a ratio of 1:4:1, and 12 mL of this mixture was added to each Teflon reaction vessel containing a sample [24–26]. Thereafter, the samples with mixed acid were heated on a graphite digestion system (ODLAB, OD-98-002P, Republic of Korea) for 1 h, and digested residues were made up to 10 mL with 1% (v/v) HNO_3_ (pH = 1–2). The final samples were stored at − 4 °C before analysis.

### Titanium distribution analysis

Inductively coupled plasma mass spectrometry (ICP–MS, ELAN DRC II, Canada) was used to measure titanium concentrations in the collected samples. Instrumental operating conditions were as follows: 1500 W of radiofrequency (RF) power, 0.9 L/min of nebulizer gas flow rate, and 1.5 L/min of auxiliary gas flow rate. Calibration standards of 1, 5, 10, 20, and 40 μg/L for titanium (1000 mg/L, Merck, Germany) were used, and coefficients of determination (R^2^) for titanium were higher than 0.999. Blank samples, which consisted of solutions without the presence of tissue, were used for the assessment of contamination during the experiments. The digestion method was applied to blank samples in order to measure the likely amounts of titanium contamination. Teflon tubes, 15 mL polypropylene tubes, and chemicals were all potential sources of titanium contamination [26]. For instrumental detection limits (IDLs), 1 mL of the lowest level of calibration standard (1 μg/L) was injected into the ICP–MS seven times (*n* = 7), and a standard deviation of analytical data was multiplied by a Student’s t value of 3.14. For method detection limits (MDLs) and the limit of quantification (LOQ), 2 mL of 5 μg/L standard was spiked into the seven blank samples, and standard deviations were multiplied by 3.14 and 10, respectively. The final volume in each blank sample was 10 mL. ICP–MS was sensitive enough to quantify all of our samples, and there was no need to improve the IDL of 0.038 μg/L. The MDLs and LOQ for the collected samples were determined to be 0.0001 mg/kg and 0.0002 mg/kg, respectively. The values presented high sensitivity and a satisfactory recovery rate (96.5 ± 2.4%). Maternal samples for non-pregnant subjects were excluded from the concentration analysis, and two samples (one fetal blood at vehicle control and one maternal liver at 100 mg/kg) were excluded from the concentration analysis because they were considered to be contaminated.

### Statistical analysis

Statistical analyses for comparisons of the various dose groups with the vehicle control group were conducted using the Pristima System (Version 7.2, Xybion Medical System Co., USA) or SAS/STAT (Version 9.4, SAS Institute Inc., USA). Litter data were statistically evaluated using the litter as a statistical unit. Multiple comparison tests for different dose groups were conducted. Continuous data were examined for variance in homogeneity using Bartlett’s Test. Homogeneous data were analyzed using analysis of variance (ANOVA), and the significance of intergroup differences was analyzed using Dunnett’s test. Heterogeneous data were analyzed using the Kruskal-Wallis test, and the significance of intergroup differences between the control and treated groups was assessed using Dunn’s rank sum test. One-way analysis of covariance (ANCOVA) was used to analyze fetal and placental weight data. The litter size was used as the covariate.

## Results

### Physicochemical characterization of TiO_2_ nanoparticles

The physicochemical characterization of TiO_2_ nanoparticles, including analyses of primary shape, primary size, purity, hydrodynamic size and zeta potential, is summarized in Table 1. The majority of the TiO_2_ nanoparticles had spherical and anatase crystal shapes with a purity of 100%. The mean primary size of the TiO_2_ nanoparticles was 17.8 ± 5.46 nm. The hydrodynamic size of the TiO_2_ nanoparticles was 341.5 nm, which indicates that TiO_2_ nanoparticles were prone to aggregation and formed a larger size in the vehicle. The zeta potential of the TiO_2_ nanoparticles in the vehicle was 35.16 mV.

**Table 1:** Physicochemical characterization of titanium dioxide nanoparticles

Physicochemical	TiO_2_ nanoparticles
Primary Shape (TEM Image)	
Primary Size	17.8 ± 5.46 nm
Purity	100%
Hydrodynamic Size	341.5 nm
Zeta Potential	35.16 mV

### Mortality and general clinical sign observation

All female rats survived through the end of the study, and no abnormal general clinical signs were observed in any group throughout the study.

### Body weights and food consumption

No test item-related changes in body weight and body weight gain were observed during the study period (Table 2). For food consumption, a statistically significant decrease during the study period (92% of control) at 1000 mg/kg was considered test item-related (Table 3). However, this decrease did not have toxicological relevance since it was minimal and there was no correlated decreased body weight or body weight gain during the study period.

**Table 2:** Body weights and body weight gain of TiO_2_ nanoparticles exposed pregnant females during the pregnancy

	TiO_2_ nanoparticles (mg/kg)			
	0	100	300	1000
Pregnant Females (N)	12	12	12	12
Body Weight (g)				
Gestation day 0	235.4 ± 8.88^a^	235.4 ± 8.93	235.6 ± 9.19	235.5 ± 9.70
Gestation day 6	275.5 ± 12.08	277.4 ± 11.41	274.5 ± 10.31	276.8 ± 13.64
Gestation day 9	288.1 ± 13.17	288.4 ± 12.59	286.6 ± 9.74	286.7 ± 14.86
Gestation day 12	307.5 ± 14.00	307.3 ± 13.20	307.6 ± 10.38	306.3 ± 18.14
Gestation day 15	323.5 ± 15.19	325.8 ± 15.92	326.7 ± 12.20	321.0 ± 19.40
Gestation day 17	342.8 ± 17.30	346.9 ± 16.35	349.2 ± 11.13	341.4 ± 18.53
Gestation day 20	389.3 ± 26.47	399.1 ± 22.46	400.1 ± 14.68	388.7 ± 21.15
Body Weight Gain (g)				
Gestation day 6–20 (treatment period)	113.8 ± 16.81	121.7 ± 15.08	125.6 ± 9.16	111.9 ± 10.30

^a^Values are presented as mean ± S.D

**Table 3:** Food consumption of TiO_2_ nanoparticles exposed pregnant females during the pregnancy

	TiO_2_ nanoparticles (mg/kg)			
	0	100	300	1000
Pregnant Females (N)	12	12	12	12
Food Consumption (g)				
Gestation day 0–6	24.3 ± 2.70^a)^	23.3 ± 1.95	24.0 ± 2.00	22.9 ± 1.55
Gestation day 6–9	26.0 ± 2.60	25.4 ± 1.97	25.7 ± 1.44	23.7 ± 1.77^*^
Gestation day 9–12	26.6 ± 2.84	24.8 ± 1.50	26.0 ± 1.35	23.5 ± 2.70
Gestation day 12–15	26.0 ± 2.19	25.6 ± 2.22	26.2 ± 1.70	24.1 ± 2.38
Gestation day 15–17	27.1 ± 1.92	26.9 ± 2.37	28.0 ± 2.12	25.6 ± 2.52
Gestation day 17–20	27.7 ± 2.43	27.1 ± 2.02	28.5 ± 2.05	26.2 ± 2.10
Gestation day 6–20 (treatment period)	26.7 ± 2.15	25.9 ± 1.73	26.8 ± 1.39	24.5 ± 2.04**

^a^Values are presented as mean ± S.D

**Significant difference at *p* < 0.01 level compared with the control group

### Organ weights and gravid uterine weight

There was no test item-related change in absolute and relative organ weights in this study (Table 4). In addition, there was no test item-related change in gravid uterine weight, corrected terminal body weight (body weight on GD 20 minus gravid uterine weight) and net body weight change (corrected terminal body weight minus body weight on GD 6) in this study (Table 5).

**Table 4:** Absolute and relative organ weights of TiO_2_ nanoparticles exposed pregnant females

	TiO_2_ nanoparticles (mg/kg)			
	0	100	300	1000
Pregnant Females (N)	12	12	12	12
Adrenal glands (g)	0.08 ± 0.010^a^	0.07 ± 0.010	0.07 ± 0.010	0.07 ± 0.009
Organ to terminal body weight ratio (%)	0.02 ± 0.003	0.02 ± 0.003	0.02 ± 0.003	0.02 ± 0.002
Brain (g)	1.86 ± 0.080	1.87 ± 0.089	1.83 ± 0.064	1.90 ± 0.045
Organ to terminal body weight ratio (%)	0.49 ± 0.047	0.49 ± 0.037	0.47 ± 0.021	0.50 ± 0.024
Heart (g)	1.09 ± 0.106	1.10 ± 0.087	1.12 ± 0.066	1.10 ± 0.103
Organ to terminal body weight ratio (%)	0.29 ± 0.028	0.28 ± 0.026	0.29 ± 0.018	0.29 ± 0.026
Kidneys (g)	2.05 ± 0.211	2.03 ± 0.160	2.06 ± 0.189	2.06 ± 0.192
Organ to terminal body weight ratio (%)	0.54 ± 0.049	0.52 ± 0.034	0.53 ± 0.047	0.55 ± 0.050
Liver (g)	14.19 ± 1.000	14.48 ± 1.039	15.16 ± 1.186	14.70 ± 0.823
Organ to terminal body weight ratio (%)	3.75 ± 0.229	3.75 ± 0.185	3.89 ± 0.237	3.88 ± 0.138
Pituitary gland (g)	0.01 ± 0.002	0.01 ± 0.003	0.01 ± 0.002	0.01 ± 0.002
Organ to terminal body weight ratio (%)	0.004 ± 0.0006	0.004 ± 0.0006	0.004 ± 0.0005	0.004 ± 0.0005
Spleen (g)	0.70 ± 0.116	0.69 ± 0.073	0.73 ± 0.115	0.71 ± 0.077
Organ to terminal body weight ratio (%)	0.19 ± 0.028	0.18 ± 0.018	0.19 ± 0.028	0.19 ± 0.019
Lung	1.35 ± 0.105	1.33 ± 0.112	1.35 ± 0.122	1.28 ± 0.107
Organ to terminal body weight ratio (%)	0.36 ± 0.032	0.35 ± 0.034	0.35 ± 0.030	0.34 ± 0.021
Right ovary (g)	0.06 ± 0.012	0.07 ± 0.01	0.06 ± 0.011	0.06 ± 0.011
Organ to terminal body weight ratio (%)	0.02 ± 0.003	0.02 ± 0.003	0.02 ± 0.003	0.02 ± 0.003
Left ovary (g)	0.06 ± 0.009	0.06 ± 0.011	0.06 ± 0.014	0.06 ± 0.012
Organ to terminal body weight ratio (%)	0.02 ± 0.002	0.01 ± 0.003	0.02 ± 0.004	0.02 ± 0.003
Thymus (g)	0.40 ± 0.084	0.37 ± 0.076	0.43 ± 0.066	0.37 ± 0.080
Organ to terminal body weight ratio (%)	0.11 ± 0.023	0.10 ± 0.019	0.11 ± 0.0175	0.10 ± 0.023

^a^Values are presented as mean ± S.D

**Table 5:** Gravid uterine weight, corrected terminal weight and net body weight change of TiO_2_ nanoparticles exposed pregnant females during the pregnancy

	TiO_2_ nanoparticles (mg/kg)			
	0	100	300	1000
Pregnant Females (N)	12	12	12	12
Gravid uterine weight (g)	76.91 ± 21.44^a^	89.31 ± 15.33	81.92 ± 6.91	78.37 ± 13.18
Corrected terminal body weight (g)^b^	312.37 ± 16.87	309.78 ± 16.54	318.14 ± 12.20	310.37 ± 20.59
Net body weight change (g)^c^	36.88 ± 11.43	32.40 ± 8.89	43.69 ± 8.34	33.54 ± 10.82

^a^Values are presented as mean ± S.D

^b^Body weight on GD 20 – Gravid uterine weight

^c^Corrected terminal body weight – Body weight on GD 6

### Cesarean section and fetal morphological examinations

There was no test item-related change in cesarean section parameters, including corpora lutea, implantation, resorptions (early and late), dead and live fetuses, sex ratio, pre-implantation loss, post-implantation loss, fetal weight, placental weight and placental macroscopic observation (Table 6). In addition, there was no test item-related change in the fetal external and visceral examinations (Table 7). In the skeletal examination, an increased ossification site of metatarsals in both hindlimbs was only observed at 100 mg/kg, but it was considered incidental since it did not have a dose response and there were no changes in other related parameters (Table 8).

**Table 6:** Caesarean section results of TiO_2_ nanoparticles exposed pregnant females during the pregnancy

	TiO_2_ nanoparticles (mg/kg)			
	0	100	300	1000
Pregnant Females (N)	12	12	12	12
Corpora lutea (N)	14.1 ± 1.83^a^	14.6 ± 1.98	13.9 ± 1.31	14.1 ± 1.56
Implantation (N)	12.8 ± 3.81	14.4 ± 2.19	13.6 ± 1.38	13.2 ± 2.55
Early resorptions (N)	0.4 ± 0.67	0.2 ± 0.58	0.4 ± 0.67	0.4 ± 0.67
Late resorptions (N)	0.0 ± 0.00	0.2 ± 0.39	0.0 ± 0.00	0.0 ± 0.00
Dead fetuses (N)	0.0 ± 0.00	0.0 ± 0.00	0.0 ± 0.00	0.0 ± 0.00
Fetal death (resorptions + dead fetuses)	0.4 ± 0.67	0.3 ± 0.65	0.4 ± 0.67	0.4 ± 0.67
Live fetuses (N)	12.4 ± 3.55	14.1 ± 2.54	13.2 ± 1.34	12.8 ± 2.45
Sex ratio (%, male)	48.1 ± 18.87	63.0 ± 14.98	45.2 ± 7.01	53.7 ± 13.75
Pre-implantation loss (%)^b^	10.0 ± 22.13	1.4 ± 3.19	2.4 ± 3.57	7.0 ± 12.07
Post-implantation loss (%)^c^	2.7 ± 4.31	2.7 ± 5.98	3.0 ± 4.65	3.0 ± 5.18
Fetal weight (g)	4.24 ± 0.25	4.38 ± 0.30	4.23 ± 0.25	4.21 ± 0.25
Covariate adjusted means	4.22	4.40	4.23	4.20
Placental weight (g)	0.50 ± 0.06	0.49 ± 0.05	0.54 ± 0.14	0.49 ± 0.05
Covariate adjusted means	0.49	0.49	0.54	0.49
Placental macroscopic observation	NAD	NAD	NAD	NAD

*NAD* No Abnormalities Detected

^a^Values are presented as mean ± S.D

^b^[(No. of corpora lutea - No. of implantation) / No. of corpora lutea] × 100

^c^[(No. of implantation – No. of live fetuses) / No. of implantation] × 100

**Table 7:** Fetal external and visceral examination results of TiO_2_ nanoparticles exposed pregnant females during the pregnancy

	TiO_2_ nanoparticles (mg/kg)			
	0	100	300	1000
Fetal External Examination				
No. of litters examined	12	12	12	12
No. of fetuses examined	149	169	158	153
Malformation				
No. of affected litters (%)	0 (0)	0 (0)	0 (0)	0 (0)
No. of affected fetuses (%)	0 (0)	0 (0)	0 (0)	0 (0)
Variation				
No. of affected litters (%)	0 (0)	0 (0)	0 (0)	0 (0)
No. of affected fetuses (%)	0 (0)	0 (0)	0 (0)	0 (0)
Fetal Visceral Examination				
No. of litters examined	12	12	12	12
No. of fetuses examined	72	81	75	74
Malformation				
No. of affected litters (%)	0 (0)	0 (0)	0 (0)	0 (0)
No. of affected fetuses (%)	0 (0)	0 (0)	0 (0)	0 (0)
Variation				
Thymus, Thymic cord				
No. of affected litters (%)	7 (58)	7 (58)	7 (58)	5 (42)
No. of affected fetuses (%)	10 (14)	13 (16)	9 (12)	7 (11)
Kidneys, Dilated renal pelvis				
No. of affected litters (%)	0 (0)	0 (0)	1 (8)	0 (0)
No. of affected fetuses (%)	0 (0)	0 (0)	2 (3)	0 (0)
Ureter, Convoluted ureter				
No. of affected litters (%)	6 (50)	6 (50)	6 (50)	7 (58)
No. of affected fetuses (%)	11 (14)	9 (11)	14 (18)	10 (14)
Ureter, Dilated ureter				
No. of affected litters (%)	7 (58)	5 (42)	4 (33)	7 (58)
No. of affected fetuses (%)	14 (18)	7 (8)	7 (9)	13 (17)

**Table 8:** Fetal skeletal examination results of TiO_2_ nanoparticles exposed pregnant females during the pregnancy

	TiO_2_ nanoparticles (mg/kg)			
	0	100	300	1000
Fetal Skeletal Examination				
No. of litters examined	12	12	12	12
No. of fetuses examined	77	88	83	79
Malformation				
No. of affected litters (%)	0 (0)	0 (0)	0 (0)	0 (0)
No. of affected fetuses (%)	0 (0)	0 (0)	0 (0)	0 (0)
Variation				
Ribs, Full thoracic supernumerary rib				
No. of affected litters (%)	0 (0)	2 (17)	0 (0)	0 (0)
No. of affected fetuses (%)	0 (0)	2 (2)	0 (0)	0 (0)
Ribs, Short thoracic supernumerary rib				
No. of affected litters (%)	2 (17)	4 (33)	3 (25)	2 (17)
No. of affected fetuses (%)	5 (6)	9 (10)	4 (5)	2 (2)
Skull, Large fontanelle				
No. of affected litters (%)	3 (25)	2 (17)	0 (0)	0 (0)
No. of affected fetuses (%)	4 (5)	2 (2)	0 (0)	0 (0)
Thoracic centrum, Asymmetric ossification				
No. of affected litters (%)	0 (0)	0 (0)	1 (8)	0 (0)
No. of affected fetuses (%)	0 (0)	0 (0)	1 (1)	0 (0)
Thoracic centrum, Bipartite ossification				
No. of affected litters (%)	0 (0)	0 (0)	1 (8)	2 (17)
No. of affected fetuses (%)	0 (0)	0 (0)	2 (2)	3 (4)
Thoracic centrum, Dumbbell ossification				
No. of affected litters (%)	2 (17)	1 (8)	0 (8)	2 (17)
No. of affected fetuses (%)	3 (3)	1 (1)	0 (0)	2 (3)
No. of ossification centers				
Sternebra	5.9 ± 0.18^a^	5.9 ± 0.20	6.0 ± 0.08	5.8 ± 0.30
Metacarpals in both forelimbs	7.8 ± 0.38	8.0 ± 0.14	8.0 ± 0.12	7.9 ± 0.12
1st phalanges in both forelimbs	1.8 ± 1.39	2.7 ± 1.20	2.1 ± 1.12	2.6 ± 1.12
Metatarsals in both hindlimbs	8.0 ± 0.09	8.3 ± 0.38^**^	8.0 ± 0.09	8.0 ± 0.03
1st phalanges in both hindlimbs	0.2 ± 0.58	0.1 ± 0.20	0.0 ± 0.06	0.0 ± 0.00
Cervical vertebra	1.3 ± 1.29	1.3 ± 1.00	1.4 ± 1.23	0.9 ± 0.86
Sacral and caudal vertebra	8.5 ± 0.45	8.7 ± 0.49	8.5 ± 0.50	8.5 ± 0.34

^a^Values are presented as mean ± S.D

^**^Significant difference at *p* < 0.01 level compared with the control group

### TiO_2_ nanoparticle distribution in tissues

**Fig. 1:**
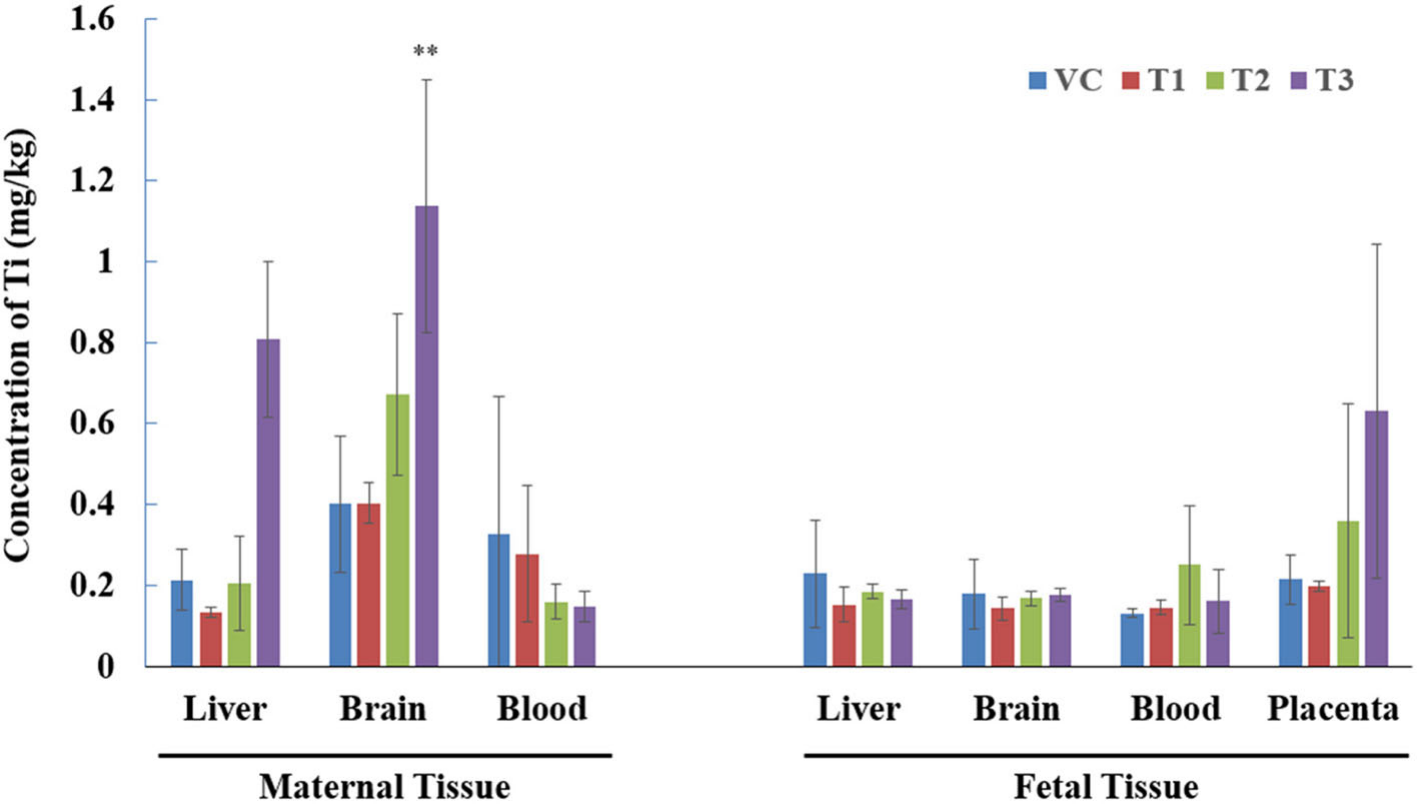
The contents of titanium in maternal and fetal tissues after orally exposed TiO_2_ nanoparticles during the pregnancy. Values are presented as mean ± S.D. (*n* = 3 or 4). VC; vehicle control, T1–3; 100, 300, and 1000 mg/kg TiO_2_ nanoparticles groups. **Significant difference at *p* < 0.01 level compared with the control group

The titanium contents were analyzed in maternal tissues (liver, brain and blood) and fetal tissues (liver, brain, blood and placenta) after the oral exposure of TiO_2_ nanoparticles during pregnancy (Fig. 1). Titanium concentrations in maternal liver, maternal brain and placenta at 1000 mg/kg were elevated compared to the concentration in control animals. In addition, at 300 mg/kg, titanium concentrations in the maternal brain and placenta were also slightly elevated. Moreover, there was no titanium concentration change in the maternal blood, fetal liver, fetal brain or fetal blood.

## Discussion and conclusion

Oral exposure to TiO_2_ nanoparticles is one of the most prevalent exposure scenarios because humans are frequently exposed to TiO_2_ nanoparticles contained in food products, liquid beverages and drugs [27, 28]. In this study, we evaluated the potential effects of oral exposure to TiO_2_ nanoparticles during pregnancy and their distribution in maternal organs as well as fetuses. TiO_2_ nanoparticles were administered by oral gavage to pregnant Sprague-Dawley rats at doses of 0, 100, 300 and 1000 mg/kg. In-life and terminal experimental endpoints, including general clinical signs, body weight changes, food consumption, macroscopic findings, organ weights, cesarean section parameters and fetal morphology, including external, visceral, and skeletal aspects, were examined. There were no TiO_2_ nanoparticle-related toxicological findings related to maternal and embryo-fetal development toxicity parameters during the study. In addition, increased titanium concentrations in the maternal liver, maternal brain and placenta were observed after high dose oral exposure during pregnancy.

The molecular mechanism of TiO_2_ nanoparticle-induced toxicity is regarded as the induction of inflammation and generation of reactive oxygen species (ROS). The accumulation of TiO_2_ nanoparticles induces chronic inflammation, which leads to the formation of ROS and cell proliferation [29]. Previous studies have demonstrated that TiO_2_ nanoparticle exposure induces the expression of inflammatory cytokines, including IL-1a, IL-1b, IL-2, IL-4, IL-6 and IL-18 [30–32]. In addition, the role of free radicals in DNA damage [27, 33], ROS-induced activation of p53-mediated DNA damage [28] and cell-derived oxidants involved in the induction of mutagenesis [34] after TiO_2_ nanoparticle exposure were investigated. Although the exact pathophysiological mechanism is not clear, these multifactorial events related to the induction of inflammation leading to the production of ROS would be the major cause in TiO_2_ nanoparticle-induced toxicity.

Experimental animal studies were also conducted to evaluate the potential effects of TiO_2_ nanoparticle exposure. An acute oral toxicity study in mice (25, 80 and 155 nm, 5000 mg/kg) reported no obvious acute toxicity, but hepatic and renal injury was observed in the histopathological examination [14]. A repeated oral toxicity study in rats (< 50 nm; 0.16, 0.4 and 1 g/kg for 14 days) revealed disturbances in metabolism and the gut microflora environment caused by slight injury to the liver and heart, as shown by urianalysis with nuclear magnetic resonance (NMR) [35]. The results from other experimental animal studies indicated that the absorption of TiO_2_ nanoparticles is able to enter the systemic circulation and induce organ injuries and inflammation [12].

Reproductive and developmental toxicity potentials of TiO_2_ nanoparticles were also reported in previous studies with zebrafish, mice and rats. In zebrafish studies, TiO_2_ nanoparticles (20 nm; 5 mg/mL, 21 nm; 0.01, 10 and 1000 μg/mL and 240–280 nm in water; 0.1 μg/mL) induced deformities in the cardiovascular system, premature hatching and impaired reproduction [36–38]. In addition, TiO_2_ nanoparticle (25 nm; 0.1 μg/mL) exposure alone did not induce toxicological effects but enhanced the metabolism of pentachlorophenol (PCP) and caused oxidative damage and developmental toxicity when co-exposed with PCP [39]. In mouse studies, subcutaneous TiO_2_ nanoparticle exposure (2570 nm; 100 μl suspended at 1 μg/μl; GDs 6, 9, 12. 15 and 25, 70 nm; 16 mg/kg; GDs 3, 7, 10 and 14) during pregnancy induced genital and cranial nerve system damage in the offspring and altered gene expression in the brain during the prenatal period [15, 16]. Intravenous TiO_2_ nanoparticle (35 nm; 0.8 mg/animal; GDs 16 and 17) exposure during pregnancy induced smaller uteri and fetuses, and TiO_2_ nanoparticles were found in the placenta, fetal liver and fetal brain [40]. In contrast, Warheit et al. [17] reported that oral exposure to different sized TiO_2_ particles (42, 43, 47, 153, 195 and 213 nm; 100, 300, and 1000 mg/kg; daily beginning on GDs 6 through 20 in rats) did not induce toxicities during pregnancy. Our study results confirmed that oral exposure to TiO_2_ nanoparticles during pregnancy did not induce toxic effects in maternal animals or embryo-fetal development endpoints. This finding is consistent with the study reported by Warheit et al. [17], although the analyzed primary particle size of the TiO_2_ nanoparticles was different from that in the previous study.

This discrepancy in reproductive and developmental results among previous studies is considered to be caused by differences in exposure routes, animal species, physicochemical properties of the nanoparticles, etc. In fact, a gastrointestinal absorption study of silver nanoparticles reported that nanoparticles were aggregated and changed their physical properties in the stomach, and the degree of these changes was especially influenced by the particle size of the nanoparticles [41]. This study indicates that oral exposure to nanoparticles is able to alleviate the toxicity by inducing the loss of characteristic properties of nanoparticles before they enter the systemic circulation when compared to directly systemically exposed routes, such as intravenous and inhalation routes. Quantitative biokinetics of TiO_2_ nanoparticle studies with oral and intravenous exposure also proved that internal exposure was much higher with intravenous than with oral exposure [42, 43]. These differences in internal exposure might result in different interactions and binding to blood proteins and biomolecules with TiO_2_ nanoparticles, which will subsequently affect uptake in organs and tissues [44]. Moreover, species differences in metabolism and placentation are considered to be important factors for birth defects occurrence [45]. In fact, the birth defect levels for selected developmental toxicants differ among animal species and humans [46].

The distribution analysis of maternal and fetal tissues in this study showed that TiO_2_ nanoparticles were retained in the maternal liver, maternal brain and placenta at 1000 mg/kg after repeated oral exposure during pregnancy. At 300 mg/kg, the TiO_2_ nanoparticle level was also slightly elevated in the maternal brain and placenta. However, it was indiscernible in the maternal blood, fetal liver, fetal brain and fetal blood of all the TiO_2_ nanoparticle-treated groups. Other studies have also reported that TiO_2_ nanoparticles can be absorbed into the systemic circulation and then distributed to internal organs. Single oral exposure of different sized TiO_2_ particles (25, 80, and 155 nm; 5 g/kg) in mice yielded retention of the particles in the liver, spleen, kidneys, brain and lung, but there was no detection of the particles in blood. The distribution level in each tissue varied depending on the TiO_2_ particle size [14]. Another single oral exposure study of TiO_2_ nanoparticles (70 nm; 30–80 μg/kg) in rats reported that the TiO_2_ particles that crossed the intestinal membrane accounted for less than 0.6% of the applied dose; however, the TiO_2_ particles were still distributed in the liver, lungs, kidneys, brain, spleen, uterus and skeleton after 7 days of exposure [43]. A single intravenous administration study of TiO_2_ nanoparticles (20–30 nm; 5 mg/kg) in rats reported that the nanoparticles were mainly retained in the liver as well as the spleen and kidney but were not detected in the blood, brain and lymph nodes [47]. A single intraperitoneal administration of TiO_2_ nanoparticles (100 nm; 324–2592 mg/kg) in mice yield retention in the spleen as well as liver, kidney and lung, but nanoparticles were not detected in the heart. The distribution level was changed depending on the sample collection time after administration [48]. These TiO_2_ nanoparticle distribution studies concluded that the liver and kidney were the most commonly observed internal organs into which the particles were distributed after they were absorbed into the systemic circulation regardless of the administration route and particle size. However, it was also concluded that the TiO_2_ nanoparticle distribution can change depending on the administration route, particle size and tissue sampling time.

One intriguing distribution result in this study was the relatively high level of TiO_2_ nanoparticles detected in the brain. Distribution studies of TiO_2_ nanoparticles in the brain have not been comprehensively conducted, but several studies have suggested that TiO_2_ nanoparticles can be deposited into the brain. Wang et al. [14] reported that acute orally exposed TiO_2_ particles (25, 80 and 155 nm; 5 g/kg) in mice were deposited in the brain and induced fatty degeneration in the hippocampus. Li et al. [49] also reported that intratracheal instillation of TiO_2_ particles (3 nm; 13.2 mg/kg, once a week for 4 weeks) in mice induced brain injury through oxidative stress. Taken together, these previous studies indicate that TiO_2_ nanoparticles are able to penetrate the blood-brain barrier, and these results were consistent with our study results.

No marked toxicities were observed in maternal animals and embryo-fetal development in this study design, but this finding does not indicate that TiO_2_ nanoparticles are completely safe during pregnancy. Generally, two species (commonly rats and rabbits) are required in this type of study to evaluate toxicity during pregnancy [50]. In fact, rats and rabbits might be able to exhibit different teratogenic results during pregnancy [45]. Moreover, it is noteworthy that TiO_2_ nanoparticles (75 nm; 10, 50 and 200 mg/kg; daily oral for 30 days) are able to induce liver edema (revealed by histopathological examination) and reductive stress (shown by biochemical assays) [51]. This result indicates that the toxicity of TiO_2_ nanoparticles can be detected by more sensitive and/or different parameters. In this regard, additional reproductive endpoints, including fertility, parturition, post-natal development and hormone analysis, were not investigated, and these parameters will support the accurate safety assessment of TiO_2_ nanoparticles. Considering the possibility of complex TiO_2_ nanoparticles exposure scenarios and their various particle sizes, it is necessary to conduct additional studies to evaluate the potential adverse effects of TiO_2_ nanoparticles.

In conclusion, we systemically investigated the maternal and embryo-fetal effects of orally exposed TiO_2_ nanoparticles during pregnancy in rats. In addition, we analyzed the titanium distribution during pregnancy using maternal and fetal tissues. As a result, there were no TiO_2_ nanoparticle-related toxicity findings in maternal animals or with respect to embryo-fetal development in this study design, and the titanium content was increased in the maternal liver, maternal brain and placenta with high-dose exposure to TiO_2_ nanoparticles. The results of this study can be used to evaluate the human risk assessment of TiO_2_ nanoparticles during pregnancy, and additional toxicity studies are considered necessary to elucidate the effects of TiO_2_ nanoparticles under various exposure scenarios and at different particle sizes.

## Data Availability

The relevant datasets supporting the conclusions of this article are included within the article, and all datasets used and analyzed during the current study are available from the corresponding author on reasonable request.

## Abbreviations

AAALAC: Association for Assessment and Accreditation of Laboratory Animal Care International
ANCOVA: Analysis of covariance
ANOVA: Analysis of variance
BET: Brunauer–Emmett–Teller
GD: Gestation day
GLP: Good laboratory practice
H_2_O_2_: Hydrogen peroxide
HF: Hydrofluoric acid
HNO_3_: Nitric acid
IACUC: Institutional Animal Care and Use Committee
ICP–MS: Inductively coupled plasma mass spectrometry
IDL: Instrumental detection limits
ILAR: Institute for Laboratory Animal Research
LOQ: Limit of quantification
MDL: Method detection limits
PCP: Pentachlorophenol
ROS: Reactive oxygen species
SPF: Specific pathogen free
TiO_2_: Titanium dioxide
